# A child with Imerslund-Gräsbeck syndrome concealed by co‐existing α-thalassaemia presenting with subacute combined degeneration of the spinal cord: a case report

**DOI:** 10.1186/s12887-021-02499-1

**Published:** 2021-01-18

**Authors:** Visvalingam Arunath, Thabitha Jebaseeli Hoole, Asanka Rathnasri, Oshanie Muthukumarana, Ishara Minuri Kumarasiri, Nishadi Dananjani Liyanage, Yasintha Costa, Sachith Mettananda

**Affiliations:** 1 Colombo North Teaching Hospital, Ragama, Sri Lanka; 2 Department of Paediatrics, Faculty of Medicine, University of Kelaniya, Ragama, Sri Lanka

**Keywords:** Imerslund-Gräsbeck syndrome, Vitamin B_12_, Thalassaemia, Inverted V sign, Subacute combined degeneration of the spinal cord, Megaloblastic changes, Hypopigmented hair, Anaemia

## Abstract

**Background:**

Imerslund-Gräsbeck syndrome is a rare genetic disease characterised by vitamin B_12_ deficiency and proteinuria.

**Case presentation:**

A 4-year old Sri Lankan boy presented with gradually worsening difficulty in walking for two weeks duration. He was previously diagnosed and managed as having non-transfusion-dependent α-thalassaemia based on the presence of hypochromic microcytic anaemia, haemoglobin H inclusion bodies in the blood film and compound heterozygous α-thalassaemia genotype with a gene deletion. However, his transfusion requirement increased over the past three months and he gradually lost his motor developmental milestones during two weeks before admission. The neurological examination revealed generalised hypotonia, exaggerated knee jerks and extensor plantar response. His complete blood count showed pancytopenia, and bone marrow biopsy revealed megaloblastic changes. Serum vitamin B_12_ and red blood cell folate levels were low. MRI revealed sub-acute combined degeneration of the spinal cord with characteristic ‘inverted V sign’. Urine analysis showed non-nephrotic range proteinuria. The diagnosis of Imerslund-Gräsbeck syndrome was made due to the presence of non-nutritional vitamin B_12_ deficiency and asymptomatic proteinuria. He showed a rapid haematological and neurological improvement to intramuscular hydroxocobalamin.

**Conclusions:**

This case report presents a rare occurrence of severe vitamin B_12_ deficiency due to Imerslund-Gräsbeck syndrome masked by co-existent α-thalassaemia, resulting in serious consequences. It highlights the need for a high index of suspicion in evaluating children with severe anaemia, especially in the presence of mixed pathologies.

## Background

Imerslund-Gräsbeck syndrome is a rare genetic disease characterised by vitamin B_12_ deficiency and proteinuria. Vitamin B_12_ deficiency in children is usually diagnosed by the presence of macrocytic anaemia before the development of neurological complications. Here we report a child with severe vitamin B_12_ deficiency due to Imerslund-Gräsbeck syndrome, whose haematological manifestations were masked by co-existing α-thalassaemia for several years, ultimately presenting with neurological manifestations.

## Case presentation

A 4-year old Sri Lankan boy presented with gradually worsening difficulty in walking and unsteadiness for two weeks duration. He was the first child of non-consanguineous parents without a significant family history. He was investigated for anaemia since 18 months of age when he presented with haemoglobin of 8.0 g/dL, mean corpuscular volume (MCV) of 57.8fL, mean corpuscular haemoglobin of 17.6 pg and hypochromic microcytic red blood cells (RBC) in the blood film. His serum ferritin, serum iron and haemoglobin high-performance liquid chromatography were normal. Peripheral blood film showed haemoglobin H inclusion bodies, and the DNA analysis revealed a compound heterozygous state for 3.7 kb α-globin gene deletion and triplicated α-globin genes (-α^3.7^/ααα). Complete blood counts of both parents were normal however, the α-globin genotype of mother revealed α-thalassaemia trait (-α^3.7^/αα). He was managed as non-transfusion dependent α-thalassaemia due to haemoglobin H disease with infrequent RBC transfusions and folic acid 1 mg daily.

During 3-months preceding the current presentation, his haemoglobin level frequently dropped below 7 g/dL thus requiring monthly blood transfusions. Then he started to lose motor developmental milestones in the two weeks before admission. At the time of admission, his gross motor development corresponded to the age of 9–12 months; he could sit unaided and stand with support, however, was not able to walk even with support. He was taking a normal balanced diet with food of both animal and plant origin and did not have any chronic illnesses.

**Fig. 1:**
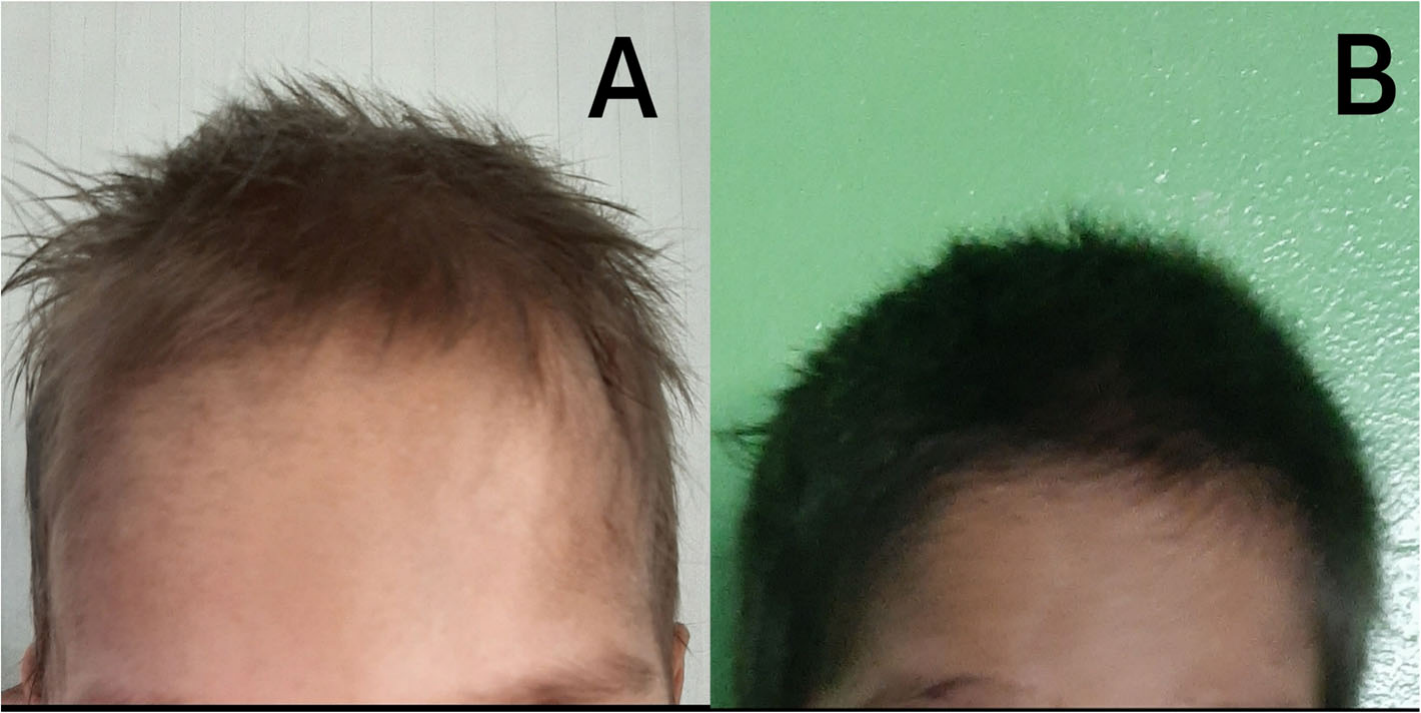
– Photographs of the child taken on admission demonstrating hypopigmented hair (**a**) and eight weeks after commencing hydroxocobalamin treatment showing improvement in hair pigmentation (**b**)

On examination, the child had low mood, poor appetite, irritability and hypopigmented hair, which were of recent onset (Fig. 1a). His weight was 11 kg (at -3SD), and the growth chart showed weight loss of 2 kg over the past six months. His height was 92 cm (between − 2SD to -3SD) and BMI was 12.9 kg/m^2^ (between − 2SD to -3SD). There were multiple petechiae in the body. Neurological examination revealed generalised hypotonia, grade 4 muscle power in lower limbs and exaggerated knee jerk and extensor plantar response bilaterally. Examination of the upper limbs was normal except for hypotonia. Glasgow coma scale and intellectual functions were unaffected. Examination of other systems was normal.

**Fig. 2:**
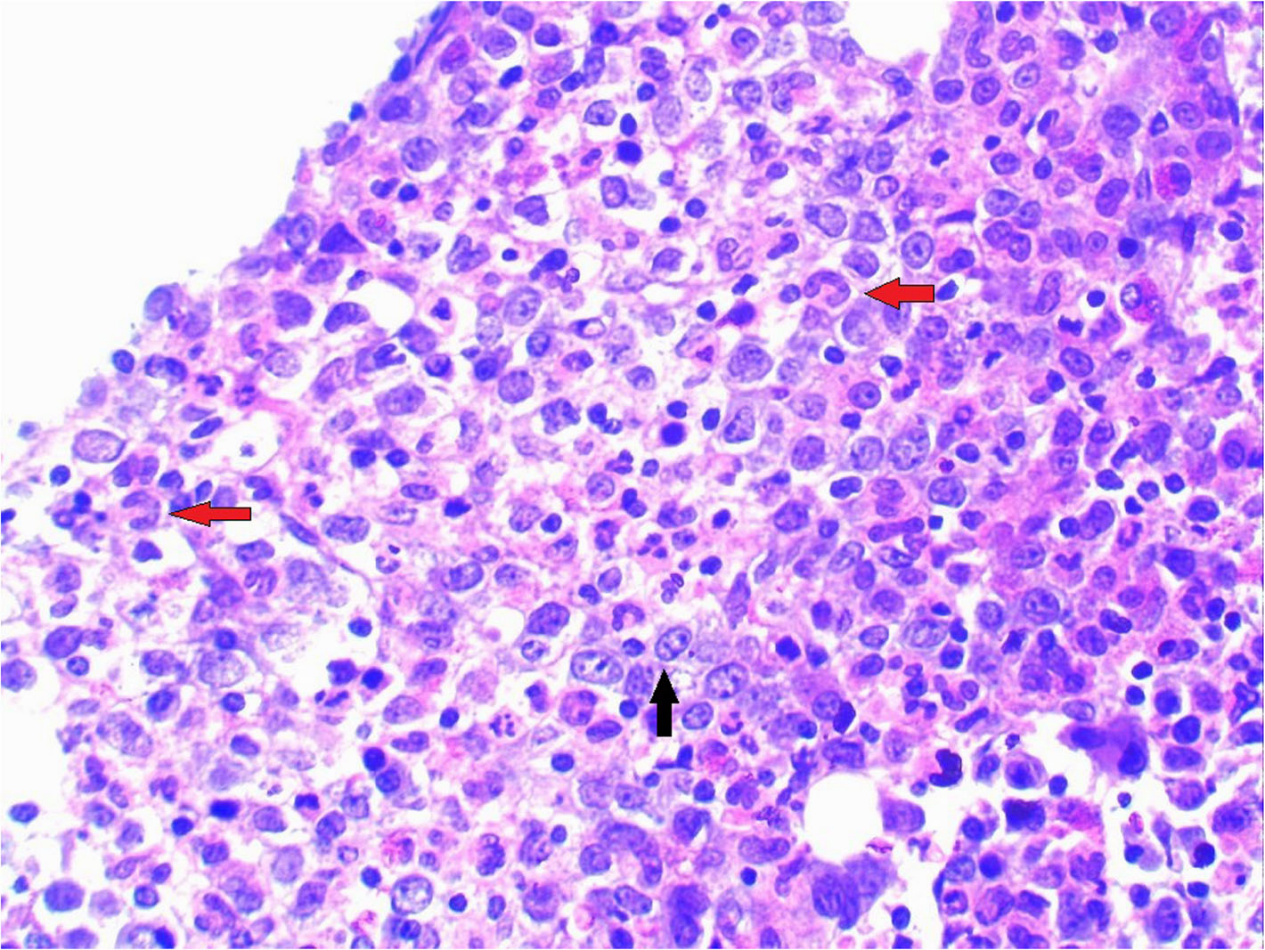
– Bone marrow trephine biopsy showing megaloblastic erythropoiesis (black arrow) with multiple giant metamyelocytes (red arrows); haematoxylin and eosin stain

His complete blood count revealed; haemoglobin- 6.8 g/dL, white cell count- 2800/mm^3^ (neutrophils-9 % and lymphocytes-89 %) and platelet- 53,000/mm^3^ (Table 1). Blood film showed pancytopenia and haemoglobin H inclusion bodies remained positive. His reticulocyte count was 0.6 %, and the bone marrow biopsy revealed mildly suppressed megaloblastic erythropoiesis with partial maturation arrest of RBC precursors. Granulopoietic progenitor cells were hypercellular with giant metamyelocytes (Fig. 2).

**Table 1:** – Haematological parameters before and after starting hydroxocobalamin treatment

	*On admission*	*Pre- treatment*	*Post-treatment*			
			***Day 3***	***Day 14***	***Day 30***	***Day 60***
Haemoglobin (g/dL)	6.8	13.4^a^	11.7	11.3	10.9	10.7
Mean corpuscular volume (fL)	90.6	93.1	86	80	73.6	63.9
White cell count (/mm^3^)	2800	5850	10,180	14,900	12,200	11,820
Neutrophil count (/mm^3^)	271	870	1425	7599	4514	4460
Platelet count (X10^3^/mm^3^)	53	19	153	691	427	493

^a^After RBC transfusion

**Fig. 3:**
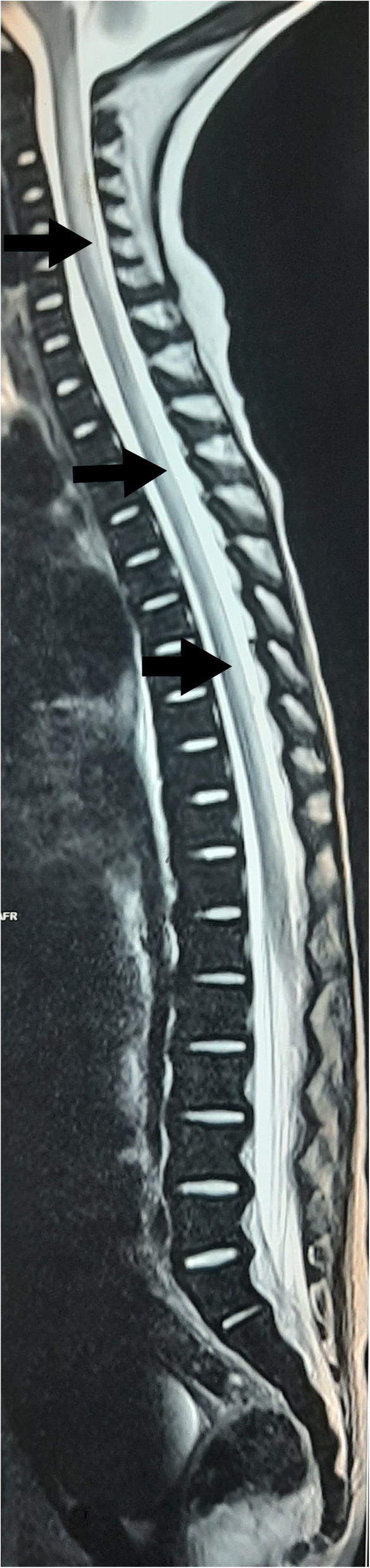
– Sagittal T2-weighted MRI image showing increased signal intensity (arrows) in posterior columns of the spinal cord extending from cervico-medullary junction to the upper border of the T12 vertebra

**Fig. 4:**
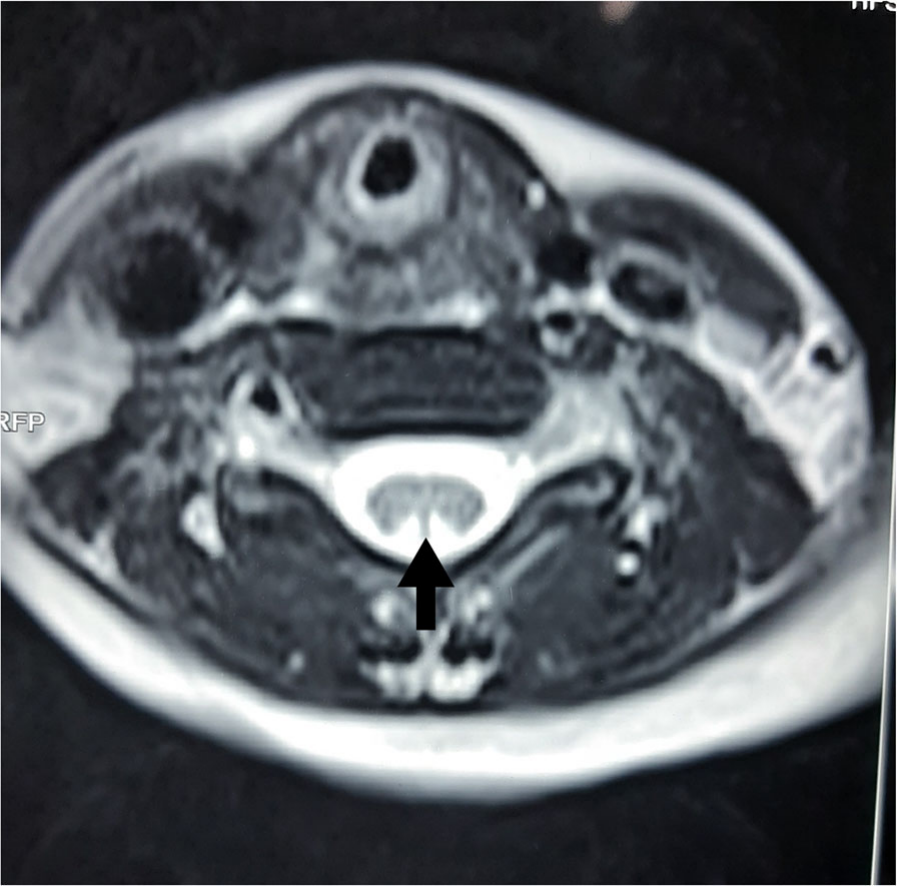
– Axial T2-weighted MRI image showing symmetrical bilateral hyperintense signal in posterior columns; the characteristic ‘inverted V sign.’

Based on the megaloblastic changes and neurological manifestations, vitamin B_12_ deficiency was suspected. Subsequent investigations revealed; low serum vitamin B_12_ (116 pg/ml; normal 187–883 pg/ml) and RBC folate (91 µg/L; normal 126–650 µg/L) levels. MRI of the spine revealed symmetrical T2-weighted high signal intensity in the posterior column from cervico-medullary junction to the 12th thoracic vertebra suggesting subacute combined degeneration of the spinal cord (Fig. 3). Axial T2-weighted images showed ‘inverted V sign’ with symmetrical bilateral hyperintense signal in posterior columns (Fig. 4). MRI of the brain revealed multiple bilateral patchy T2-weighted high signal intensity in the centrum semiovale and deep white matter areas with mild cerebral and cerebellar atrophy. The ventricular system, extra-axial cerebrospinal fluid spaces and cerebellar folia were prominent. His nerve conduction study was normal, anti-intrinsic factor antibody titre was 14.7U (Normal < 20.0), and anti-parietal cell antibodies were negative. Urine analysis revealed urine albumin 2 + with normal microscopy. Urine protein: creatinine ratio was 225 mg/mmol suggesting non-nephrotic range proteinuria. His liver and renal function tests, serum electrolytes, serum calcium, serum protein and renal ultrasonography were normal; however, 25-hydroxy vitamin D level was 27.3 ng/mL (normal 30–100 ng/mL).

Due to the presence of non-nutritional vitamin B_12_ deficiency and proteinuria in the absence of renal pathology, the diagnosis of Imerslund-Gräsbeck syndrome was made. Genetic testing was not done due to its unavailability locally and difficulties in getting it done internationally. He was commenced on intramuscular hydroxocobalamin 1 mg daily for two weeks followed by 1 mg weekly for eight weeks and then 1 mg monthly. With treatment, he demonstrated rapid improvement of general well-being, hair pigmentation, neurological functions, and haematological manifestations over 4–8 weeks (Fig. 1b; Table 1). He could walk unaided after two weeks and was transfusion independent after two months.

## Discussion and conclusions

Imerslund-Gräsbeck syndrome is an autosomal recessive genetic disease characterised by vitamin B_12_ deficiency and proteinuria [1]. The primary pathology is selective malabsorption of vitamin B_12_ due to a defect in the receptor for vitamin B_12_-intrinsic factor complex in the ileal enterocyte. Mutations in the cubilin gene (*CUBN*) and amnionless gene (*AMN*) which code for protein components of this receptor are known to cause Imerslund-Gräsbeck syndrome [2].

The deficiency of vitamin B_12_ leads to haematological and neurological complications [3]. The classic neurological manifestations are due to subacute combined degeneration of the posterior and lateral columns of the spinal cord, which occur in long-standing deficiency. These manifestations are rare at present due to early recognition of vitamin B_12_ deficiency following haematological manifestations that include macrocytic RBCs and megaloblastic anaemia.

Our patient was investigated for anaemia for over two years. Due to microcytic RBC and features of haemoglobinopathy with haemoglobin H disease, he was diagnosed to have haemoglobin H disease, a subtype of α-thalassaemia. However, his α-globin genotype showed deletion of one α-globin gene that is consistent with a silent α-thalassaemia carrier. So, blood smear should be normal. Also, very few haemoglobin H inclusion bodies may be rarely found in α-thalassemia trait. Therefore, it is likely that his anaemia was not due to haemoglobin H disease but due to vitamin B_12_ deficiency of which the RBC phenotype was masked by the co-existent α-thalassaemia trait.

The severe nature of vitamin B_12_ deficiency in our patient despite normal dietary intake was suggestive of a genetic cause. The diagnosis of Imerslund-Gräsbeck syndrome was confirmed by demonstrating proteinuria and normal renal functions. Proteinuria is a recognised feature of Imerslund-Gräsbeck syndrome due to *CUBN* mutations. This is because cubilin plays an important role in the reabsorption of filtered albumin from proximal renal tubule [4]. In addition, mutations in *CUBN* is associated with abnormal vitamin D metabolism due to the action of cubilin in the reabsorption of vitamin D binding protein from glomerular filtrate [5]. Our patient had low levels of 25-hydroxy vitamin D supporting the diagnosis of Imerslund-Gräsbeck syndrome due to *CUBN* mutation.

A review of the literature revealed few previous reports of patients with concurrent megaloblastic anaemia and thalassaemia and one patient with co-existing Imerslund-Gräsbeck syndrome and α-thalassaemia [6, 7]. All these patients showed some features of macrocytic anaemia in the peripheral blood film, and none of them developed neurological manifestations. In contrast, blood film of our patient was compatible with α-thalassaemia without macrocytosis. Therefore, the diagnosis was only made when the child presented with subacute combined degeneration of the spinal cord.

Another unusual feature in this child was the presence of hypopigmented hair. Although skin hyperpigmentation is a well-recognised dermatological manifestation of vitamin B_12_ deficiency, the presence of hypopigmented hair is extremely rare with only a few previous reports [8, 9]. The mechanism of this is still unclear; however, it is likely to be related to vitamin B_12_ deficiency because it showed a rapid response to treatment.

This case report presents a rare association of Imerslund-Gräsbeck syndrome and α-thalassaemia. It also highlights that vitamin B_12_ deficiency can be concealed by co-existent thalassaemia resulting in severe consequences. Therefore, a high index of suspicion is needed in evaluating a child with severe anaemia, especially in the presence of mixed pathologies.

## Data Availability

Not applicable.

## Abbreviations

MCV: Mean corpuscular volume
RBC: Red blood cells
MRI: Magnetic resonance imaging
*CUBN*: Cubilin gene
*AMN*: Amnionless gene
